# The Effect of Ratio of Changing to Static Stimuli on the Attentional Capture

**DOI:** 10.1038/s41598-018-35743-3

**Published:** 2018-11-28

**Authors:** Fuminori Ono

**Affiliations:** 1 Yamaguchi University, Yamaguchi, Japan; 2 The Research Institute for Time Studies, Yamaguchi, Japan

## Abstract

Studies have shown that appearing or disappearing objects attract more attention than static objects. This study examined the modulation of attention attracted by transient signals by systematically manipulating the ratio of changing (appearing/disappearing) to static stimuli. The results revealed that the effect of transient stimuli in attracting attention was diminished by simultaneously appearing (disappearing) peripheral stimuli and that the position where nothing was presented (the remaining stimulus) attracted attention when the number of appearing (disappearing) peripheral stimuli was increased. These findings suggest that the sudden change does not always capture attention, and whether changed things are attended or unchanged things are attended is determined depending on the proportion of things that change and do not change.

## Introduction

If an object suddenly appears in our field of view, a quick and flexible orientation of visual attention to that object is generally caused. Allocation of attention in this way helps obtain important information about the object (shape, color, size, and so on) so that we can respond properly and promptly. There is substantial evidence to suggest that the sudden appearance of objects is particularly effective in attracting attention^1,2^. Posner’s attentional paradigm requires participants to detect a peripheral target preceded by an onset of a cue stimulus. If the cue position matches the target position, reaction time has been found to faster than when the two positions are different. This facilitatory effect of the onset has become known as the attentional cueing effect, and is thought to occur because the onset cue automatically captures attention^3–5^.

Two competing hypotheses have been proposed to explain how attentional capture responds to the sudden appearance of an object. Under the new object hypothesis, an onset object captures attention in virtue of its novelty^6–8^. The appearance of an object introduces change to the visual environment and forces an immediate update of visual short term memory (VSTM). To accomplish this, an attentional interrupt is triggered and the representation of the new object can be reflected in the VSTM. In this way, the new object is attentionally prioritized and thus “captures” attention. Under the transient hypothesis, attention is drawn by sudden sensory transients that occur when an object undergoes noticeable change^1,2^. For example, when an object moves into view, the motion transients generated by the object will capture attention^9,10^. In this account, it is the motion signal that draws attention, not the fact that a new object is presented in our field of view. Many theories of attentional capture have been tested in experiments aiming to establish the factors to which attention is directed by local and distinct changes in the environment. In other words, these theories rely on experimental paradigms that introduce new, local, and distinct changes in the visual environment to determine what type of change attracts attention to its position. Hilchey, Taylor, & Pratt^11^ refer this paradigm as the ‘new-event approach’.

Previous studies have examined the extent to which the onset stimulus induces attentional capture using a visual search task^8,12^. In their visual search task, the stimulus is first presented with an array of figure-eight pre-masks. The characters of the search display are formed by removal of some segments from each premask (non-onset stimulus). An additional character appears in the blank position (onset stimulus). It has been shown experimentally that the reaction time to the onset target character does not depend on the number of presented characters, but the response time for the non-onset target increases with the number of characters. This indicates that the changing stimulus (onset stimulus) is attended first when presented among the non-changing stimuli (non-onset stimuli) and is considered as evidence of capturing attention. Recently, Hilchey, Taylor, & Pratt^11^ demonstrated that an old stimulus with no change can attract attention using a modified additional singleton paradigm. In their study, following a preview array of placeholder stimuli, one placeholder stimulus is converted to the target stimulus, while all the other placeholders except for one change in luminance. This static singleton location, with no new stimulus or sensory transient, produces a clear pattern of attentional capture near that location (see also^13,14^). This finding indicates that the static stimulus is attended first when presented among the changing stimuli. Taken together, these findings imply that the effect of attentional capture may change depending on the relative amount of changing stimuli and static stimuli. Therefore, this study directly examined the modulation of attention through changing and static signals by systematically manipulating the ratio of changing to static stimuli, and identifies factors that the static stimuli attract attention.

**Figure 1:**
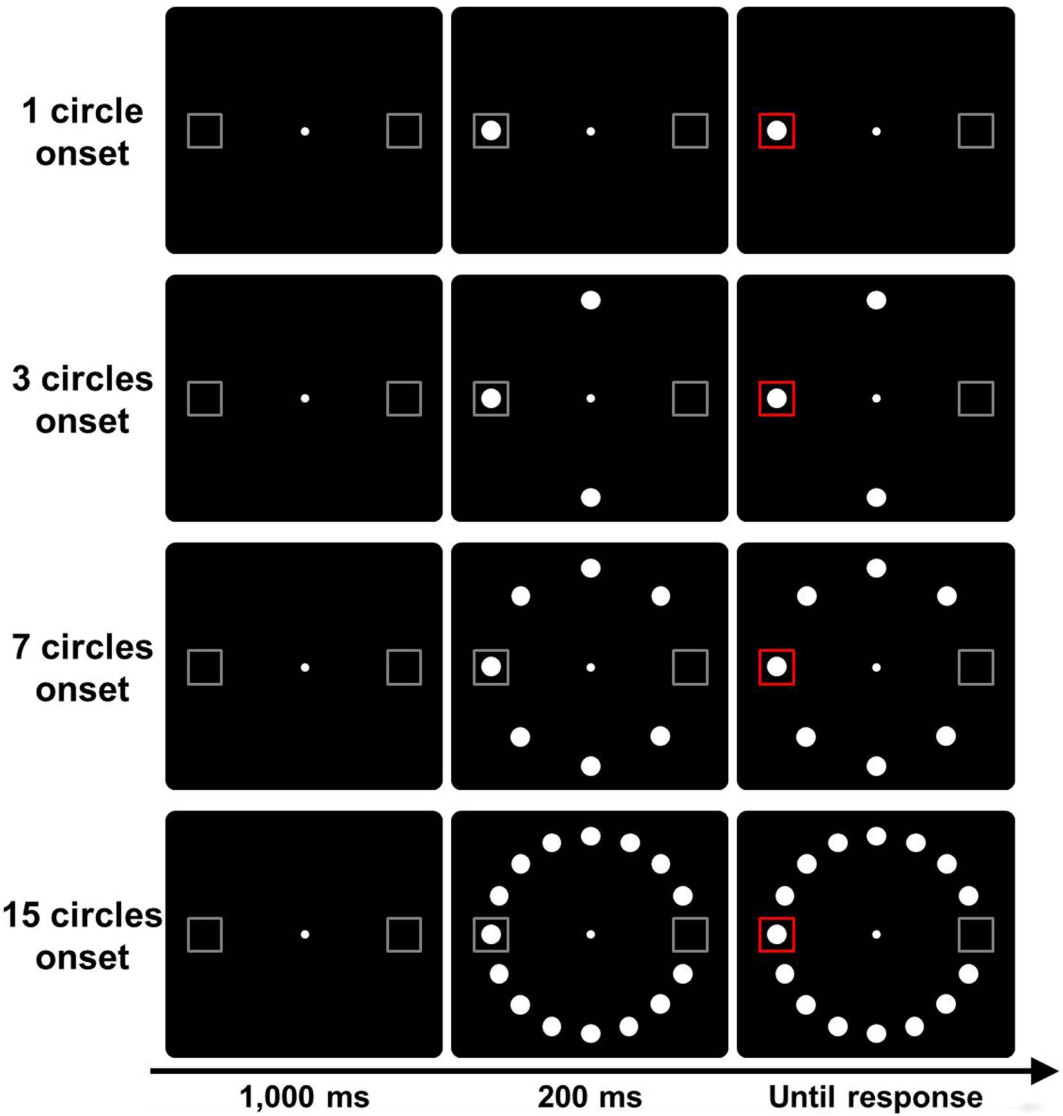
Trial sequence used in Experiment 1. The target was the change in color (gray to red) of either the left or right placeholder boxes. A trial in which the target was presented at the location where the circle appeared was considered to be a change trial.

In Experiment 1, each trial consisted of a peripheral target preceded by additional circles. The sequence of events is shown in Fig. 1. In order to examine the effect of the ratio of changing to static stimuli, the numbers of circles presented in the additional display were manipulated.

## Results and Discussions

### Experiment 1: Onset stimuli

**Figure 2:**
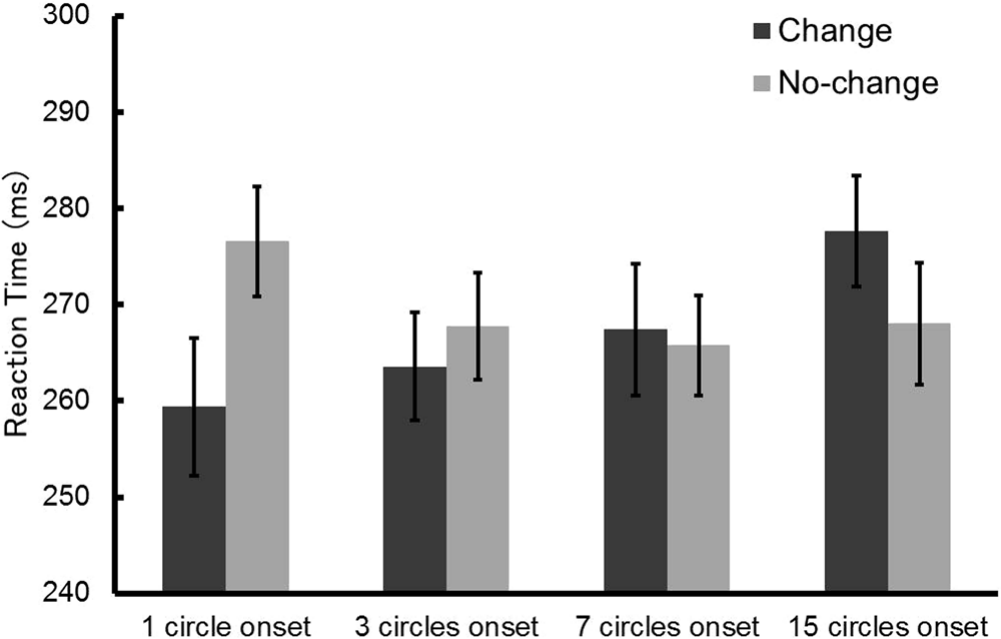
Mean reaction times in Experiment 1. Error bars show standard errors of the mean.

For purposes of analysis, a trial in which the target was presented at the location where the circle appeared was considered to be a change trial, and a trial where a target was presented at the location where the circle did not appear was considered to be a no-change trial. Error trials were excluded from the analysis (1.9% of all trials). The mean reaction times are presented in Fig. 2. Planned comparisons showed that, in the 1 circle onset condition, the reaction times of change trials were significantly shorter than that of no-change trials (*t*(15) = 3.37, *p* = 0.004, *d* = 0.69). This result is consistent with previous literature, in that the sudden appearance of an object attracted attention (e.g.,^1,2^). In the 3 circles onset and 7 circles onset conditions, there was no significant difference between change and no-change trials (*t*(15) = 0.90, *p* = 0.38, *d* = 0.19; *t*(15) = 0.32, *p* = 0.76, *d* = 0.07). In the 15 circles onset condition, the reaction times of change trials were significantly longer than that of no-change trials (*t*(15) = 2.44, *p* = 0.03, *d* = 0.41). This result is also consistent with previous literature, in that the position where nothing was presented attracted attention^11,14,15^. These findings suggest that attentional capture changed depending on the ratio of changing to static stimuli.

### Experiment 2: Offset stimuli

**Figure 3:**
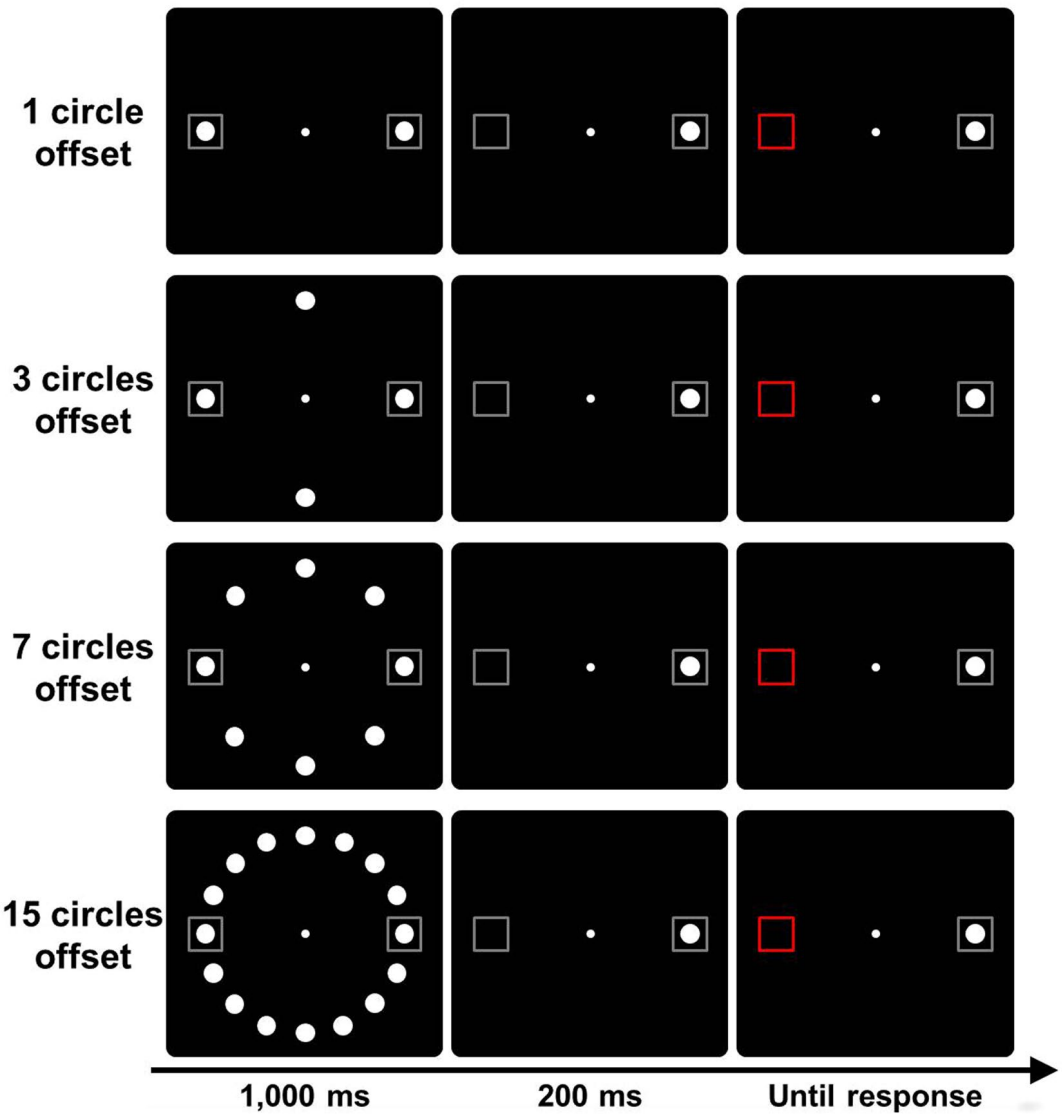
Trial sequence used in Experiment 2. The target was the change in color (gray to red) of either the left or right placeholder boxes. A trial in which the target was presented at the location where the circle disappeared was considered to be a change trial.

Only a few studies have investigated the effect of offset compared to onset cues. For example, Pratt and McAuliffe^16^ compared the effects of a single onset cue, a single offset cue, and simultaneous onset and offset cues (opposite positions). The results showed that the onset and offset cues provided equivalent facilitation effects. This finding suggests that the offset cues are handled in the same way as the onset cues by the attentional system. In Experiment 2, a trial consisted of a peripheral target preceded by initial circles and offset of the circles. The sequence of events is shown in Fig. 3. In order to examine the effect of the ratio of changing to static stimuli, the numbers of circles presented in the initial display and erased in the second display were manipulated.

**Figure 4:**
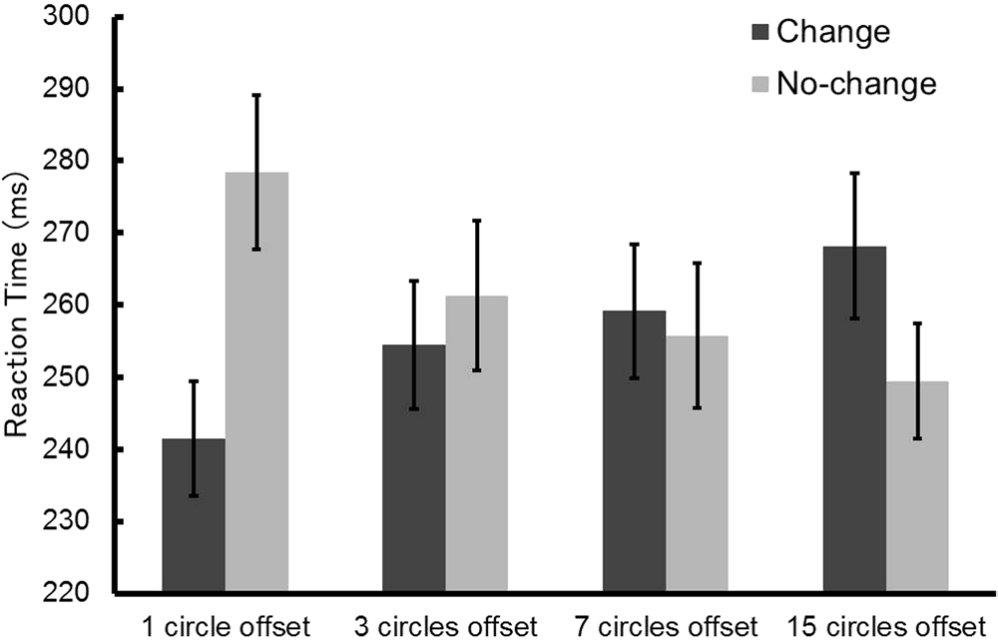
Mean reaction times in Experiment 2. Error bars show standard errors of the mean.

For purposes of the analysis, a trial in which the target was presented at the location where the circle disappeared was considered to be a change trial, and a trial where a target was presented at the location where the circle remained was considered to be a no-change trial. Error trials were excluded from the analysis (3.0% of all trials). The mean reaction times are presented in Fig. 4. Planned comparisons showed that, in the 1 circle offset condition, the reaction times of change trials were significantly shorter than that of no-change trials (*t*(15) = 7.51, *p* < 0.001, *d* = 1.01). This result is consistent with previous literature, in that the sudden disappearance of an object attracted attention^16,17^. In the 3 circles offset and 7 circles offset conditions, there was no significant difference between change and no-change trials (*t*(15) = 0.98, *p* = 0.34, *d* = 0.18; *t*(15) = 0.60, *p* = 0.56, *d* = 0.09). In the 15 circles offset condition, the reaction times of change trials were significantly longer than that of no-change trials (*t*(15) = 4.52, *p* < 0.001, *d* = 0.53). This result is also consistent with previous literature, in that the remaining stimulus without any change attracted attention^11,13,14^. These findings further suggest that the effect of attentional capture changed depending on the ratio of changing to static stimuli.

## General Discussion

The results of the two experiments indicate that the effect of transient signals in attracting attention can be modulated by systematically manipulating the ratio of changing to static stimuli. Experiment 1 revealed that the effect of onset stimuli in attracting attention was diminished by simultaneously presented peripheral stimuli, and that the position in which nothing was presented attracted attention when the number of presented peripheral stimuli was increased. The results of Experiment 2 revealed that the effect of offset stimuli in attracting attention was diminished by simultaneously disappearing peripheral stimuli, and that the remaining stimulus attracted attention when the number of disappearing peripheral stimuli was increased. Note that the stimuli array from the time immediately disappearing (Experiment 2) of circle stimuli was the same under all the conditions. Therefore, the effect observed in the present study is attributed to the difference of stimuli array in the initial and second displays.

As noted earlier, the hypotheses of attentional capture rely on experimental paradigms that introduce new, local, and distinct changes in the visual environment to determine what type of change attracts attention to its position. Consequently, the existing hypotheses cannot explain the effects found in the present study. For example, under the new object hypothesis, an onset object captures attention in virtue of its novelty^6–8^. Under the transient hypothesis, attention is drawn by sudden sensory transients that occur when an object causes a noticeable change (e.g.,^1,2^). However, in the present study, the position in which nothing was presented (Experiment 1) and the static stimulus without any change (Experiment 2) attracted visual attention.

With regard to the possible mechanisms through which attentional capture is produced, von Muhlenen, Rempel, and Enns^18^ propose the unique event hypothesis which states that a local alteration to the environment is most likely to capture attention when all other stimulus locations remain static (see also^19^). However, this hypothesis alone cannot explain the results observed in the present study. The effects of attentional capture were reversed depending on whether the number of changing stimuli was more than that of the static stimuli. To explain the results of present study, I propose whether changed things are attended or unchanged things are attended is determined depending on the proportion of things that change and do not change. That is, in situations with a large number of change (no-change) stimuli, one no-change (change) stimulus attracts visual attention.

This proportion account might be able to explain the capacity of attentional capture. In previous studies^20–22^, the capacity of attentional capture has been investigated by examining the attenuation of attentional capture when presenting multiple onsets with the target. Yantis and Johnson^20,21^ showed that up to three or four abrupt onsets are automatically prioritized, but Sunny and von Mühlenen^21^ showed that only one onset is automatically prioritized. Although the number of prioritized onsets was different, these findings suggest that the attentional capture was abolished when multiple onsets are presented. Sunny and von Mühlenen^22^ concluded that attentional capture is triggered by an increased saliency signal. An alternative (but not exclusive) possibility is that the capacity of attentional capture might be due to proportion of things that change and do not change.

In conclusion, the present studies show that the effect of attentional capture changes depending on the difference in configuration between the initial and second stimulus displays. This suggests that the ratio of a display’s salience may contribute to the attentional capture of vision. In this study, I manipulated the number of changing and static stimuli. However, I am able to manipulate other properties of visual stimuli, such as the density, proximity, and spatial arrangement. The effects of these properties on the attentional capture warrant further study in the future.

## General Methods

### Ethics statement

All the experiments in this paper were approved by the ethical committee in Yamaguchi University (2015–007) and conducted according to the principles of the Declaration of Helsinki. Written informed consent was obtained from all participants.

### Materials

Stimuli were programmed in MATLAB using the Psychophysics Toolbox extensions^23,24^, and were viewed on a CRT monitor. The viewing distance was approximately 60 cm.

### Experiment 1

#### Participants

Sixteen paid participants were recruited and participated in the experiment. The number of participants was determined on the basis of the research of Yantis and Hillstrom^8^. All participants had normal or corrected-to-normal vision (aged 19–39 years; seven female).

#### Stimuli and Procedure

The basic sequence was identical to Experiment 1 of Pratt and McAuliffe^16^. All stimuli appeared against a black background on the screen. Participants initiated each trial by pressing the space bar on a computer keyboard. Following that, the initial display was presented for 1,000 ms. The initial display consisted of a central fixation dot and two placeholder boxes. The central fixation dot was presented at center of the display and was 0.1° in diameter, presented in white. The placeholder boxes were located on the horizontal meridian to the left and right of a central fixation dot. The placeholder boxes were centered 3.8° from fixation dot and were 1.1° square, presented in gray. The circle stimuli consisted of white discs, 1.1° in diameter. The circles were placed at equal distances on an imaginary circle with a radius of 3.8°.

After the initial display of 1,000 ms, the additional circles appeared. The number of additional circles depended on the condition. In the ‘1 circle onset’ condition, one circle was presented in either the right or left placeholder boxes. In the ‘3 circles onset’ condition, two circles were added to the one circle in the 1 circle onset condition, and a total of three circles were presented. In the ‘7 circles onset’ condition, four circles were added to the three circles in the 3 circles onset condition, and a total of seven circles were presented. In the ‘15 circles onset’ condition, eight circles were added to the seven circles in the 7 circles onset condition, and a total of fifteen circles were presented.

The target was presented 200 ms after the appearance of the additional circles. The target was the change in color (gray to red) of either the left or right placeholder boxes. The participants judged which of the left and right boxes turned red, and responded by pressing the left or the right key, respectively. The participants were instructed to respond as quickly as possible while attempting to minimize errors. By pressing the key, the target and the circles disappeared, and the next trial started by pressing the space bar.

### Design

Each participant completed 16 practice and 256 test trials (two blocks of 128 trials). There were 32 change and 32 no-change trials for each of the four conditions (1 circle onset, 3 circles onset, 7 circles onset, and 15 circles onset). The locations of the target and the appearing circle in the placeholder box were randomized across the experiment. If the participants pressed the incorrect key or responded faster than 100 ms or slower than 1,000 ms, the response was considered an error. Error trials were not repeated.

### Experiment 2

#### Participants

Sixteen paid participants were recruited and participated in the experiment. All participants had normal or corrected-to-normal vision (aged 19–39 years; five female).

#### Stimuli and Procedure

The trial sequence was similar to that used in Experiment 1. The number of circles in the initial display depended on the condition. In the ‘1 circle offset’ condition, two white circles were presented in the right and left placeholder boxes. In the ‘3 circles offset’ condition, the four circles were presented. In the ‘7 circles offset’ condition, the eight circles were presented. In the ‘15 circles offset’ condition, the sixteen circles were presented. After the initial display of 1,000 ms, the circles disappeared in the second display, leaving one of the left and right circles. The target was presented 200 ms after the disappearance of the circles. Note that the stimuli array from the time immediately after the disappearing of circles was the same under all the conditions.

#### Design

Each participant completed 16 practice and 256 test trials (two blocks of 128 trials). There were 32 change and 32 no-change trials for each of the four conditions (1 circle offset, 3 circles offset, 7 circles offset, and 15 circles offset).

## References

[1] Franconeri SL, Hollingworth A, Simons DJ (2005). Do new objects capture attention?. Psychol. Sci..

[2] Yantis S, Jonides J (1984). Abrupt visual onsets and selective attention: Evidence from visual search. J. Exp. Psychol. Hum. Percept. Perform..

[3] Posner MI (1980). Orienting of attention. Q. J. Exp. Psychol..

[4] Posner, M. I. & Cohen, Y. Components of visual orienting. In Bouma, H. & Bouwhuis, D. G. (eds) Attention and performance X: Control of language processes. 531–556 (Hillsdale, NJ: Erlbaum, 1984).

[5] Yantis S, Jonides J (1990). Abrupt visual onsets and selective attention: Voluntary vs. automatic allocation. J. Exp. Psychol. Hum. Percept. Perform..

[6] Davoli CC, Suszko JW, Abrams RA (2007). New objects can capture attention without a unique luminance transient. Psychon. Bull. Rev..

[7] Rauschenberger R (2003). Attentional capture by auto-and allo-cues. Psychon. Bull. Rev..

[8] Yantis S, Hillstrom AP (1994). Stimulus-driven attentional capture: evidence from equiluminant visual objects. J. Exp. Psychol. Hum. Percept. Perform..

[9] Abrams RA, Christ SE (2005). Onset but not offset of irrelevant motion disrupts inhibition of return. Percept. Psychophys..

[10] Franconeri SL, Simons DJ (2003). Moving and looming stimuli capture attention. Percept. Psychophys..

[11] Hilchey MD, Taylor JET, Pratt J (2016). Much ado about nothing: Capturing attention toward locations without new perceptual events. J. Exp. Psychol. Hum. Percept. Perform..

[12] Yantis S, Jonides J (1996). Attentional capture by abrupt onsets: new perceptual objects or visual masking?. J. Exp. Psychol. Hum. Percept. Perform..

[13] Pinto Y, Olivers CNL, Theeuwes J (2008). Static items are automatically prioritized in a dynamic environment. Vis. Cognit..

[14] Taylor JET, Hilchey MD, Pratt J (2018). Out with the new, in with the old: Exogenous orienting to locations with physically constant stimulation. Psychon. Bull. Rev..

[15] Kiss M, Eimer M (2011). The absence of a visual stimulus can trigger task set independent attentional capture. Psychophysiology.

[16] Pratt J, McAuliffe J (2001). The effects of onsets and offsets on visual attention. Psychol. Res..

[17] Riggio L, Bello A, Umiltà C (1998). Inhibitory and facilitatory effects of cue onset and offset. Psychol. Res..

[18] von Mühlenen A, Conci M (2016). The role of unique color changes and singletons in attention capture. Atten. Percept. Psychophys..

[19] von Mühlenen A, Rempel MI, Enns JT (2005). Unique temporal change is the key to attentional capture. Psychol. Sci..

[20] Yantis S, Jones E (1991). Mechanisms of attentional selection: temporally modulated priority tags. Percept. Psychophys..

[21] Yantis S, Johnson DN (1990). Mechanisms of attentional priority. J. Exp. Psychol. Hum. Percept. Perform..

[22] Sunny MM, von Mühlenen A (2013). Attention capture by abrupt onsets: re-visiting the priority tag model. Front. Psychol..

[23] Brainard DH (1997). The psychophysics toolbox. Spat. Vis..

[24] Pelli DG (1997). The VideoToolbox software for visual psychophysics: transforming numbers into movies. Spat. Vis..

